# Acute unilateral proptosis in childhood: suspect myeloid sarcoma

**DOI:** 10.22336/rjo.2021.17

**Published:** 2021

**Authors:** Manpreet Singh, Sagarika Snehi, Pulkit Rastogi, Kalaivani Jayakumar, Manpreet Kaur, Pankaj Gupta

**Affiliations:** * Department of Ophthalmology, Post Graduate Institute of Medical Education and Research, Chandigarh, India; ** Department of Histopathology, Post Graduate Institute of Medical Education and Research, Chandigarh, India

**Keywords:** myeloid sarcoma, childhood proptosis, proptosis, acute myeloid leukemia, acute proptosis

## Abstract

As the first and only presenting feature of acute myeloid leukemia (AML), unilateral proptosis in children is uncommon. We report the cases of two girls who had no systemic clinical manifestations of AML. Orbital imaging showed space-occupying infiltrating lesions without surrounding bone erosion. Incisional biopsy and immunohistochemistry were diagnostic for myeloid sarcoma. Systemic workup and bone marrow examination showed features of AML. Systemic chemotherapy was administered to both children, who responded well to the treatment. Myeloid sarcoma should be kept in the differentials of the children presenting with isolated proptosis. Immunohistochemistry may provide an accurate diagnosis and early treatment may lead to a prompt recovery with a good prognosis.

## Introduction

Myeloid sarcoma (MS) is an extramedullary infiltration of leukemic cells in patients having myelodysplastic syndromes. MS can be associated with acute myeloid leukemia (AML) in around 2.5-8% of the cases [**1**]. It is also known as chloroma, myelosarcoma, myelocytoma, and chloroleukemia. MS can involve any body part; however, the orbit is a relatively uncommon location. It presents as rapidly progressive, usually bilateral proptosis in children with existing AML [**1**]. As the first and only clinical presentation of AML, proptosis has been less commonly reported [**1**-**4**].

The differential diagnosis for a rapidly growing unilateral proptosis in the pediatric age group includes inflammatory (orbital cellulitis) and malignant etiologies. Rhabdomyosarcoma is the most common malignant etiology, followed by metastatic neuroblastoma, lymphoma, and myeloid sarcoma [**1**,**2**].

We report two cases of MS presenting with unilateral proptosis, without clinical evidence of systemic dysfunction. Orbital imaging, incision biopsy, and immunohistochemistry (IHC) helped diagnose myeloid sarcoma due to systemic AML. Our research article followed the tenets of the Declaration of Helsinki (1975), as revised in 2000 and 2008. A written informed consent was obtained from the parents of our patients to publish their clinical details and non-identifiable images in the scientific journals. 

## Case report

**Fig. 1:**
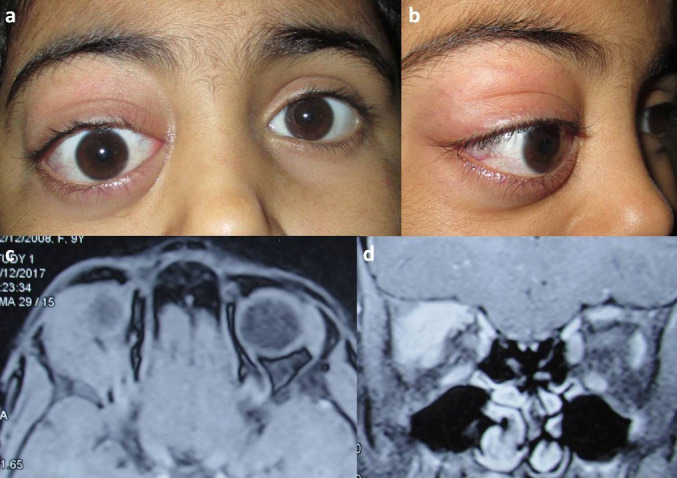
**a** Primary gaze - OD shows inferior dystopia with conjunctival congestion; **b.** The lateral view shows supero-temporal sulcus fullness, proptosis, and conjunctival congestion **c.** MRI (axial, T2W, superior) shows a hyperintense soft-tissue lesion in supero-temporal orbit (lacrimal gland region) with extension into posterior orbit; **d.** MRI (coronal, posterior) soft-tissue lesion in the extraconal compartment with an infero-medial displacement of orbital contents i.e., extraocular muscles

*Case 1*: A 9-year-girl had a sudden onset, painless protrusion OD of 4 months duration. There was no history of redness, diminution of vision, fever, malaise, or weight loss. She was diagnosed as right dacryoadenitis elsewhere and showed a good response with oral steroids (1mg/ Kg). However, the proptosis worsened with the tapering of steroids. On presentation, her visual acuity OU was 6/ 6, and intraocular pressures were normal. The OD was displaced down and outwards (4 mm) with superior orbital sulcus fullness and limited elevation (**Fig. 1a,b**). Palpation showed a non-tender, hard mass (18 mm×12 mm) in the right supero-temporal orbit. The pupillary reactions and the rest of the ophthalmic examination were unremarkable.

**Fig. 2:**
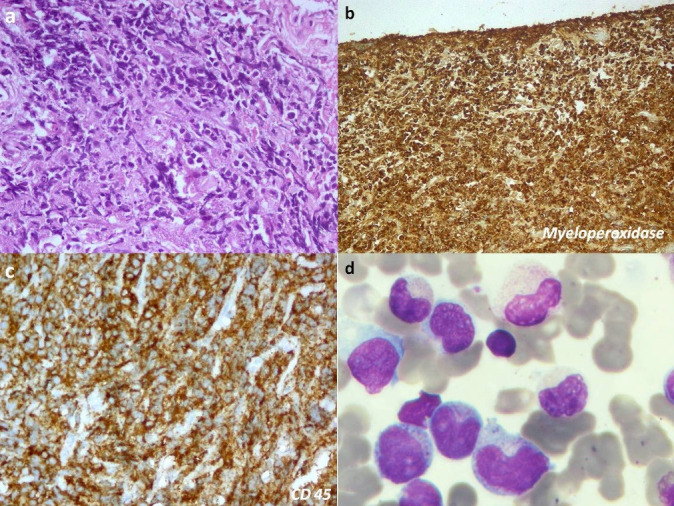
**a** (H&E, 100x) Small round-blue tumor cells arranged in diffuse sheets, cells having fine round-oval nuclei with fine chromatin, prominent nucleoli, and moderate cytoplasm; **b,c.** IHC showing positivity for myeloperoxidase (MPO) and CD45; **d.** Bone marrow aspirate showing blast cells with premature configuration

**Fig. 3:**
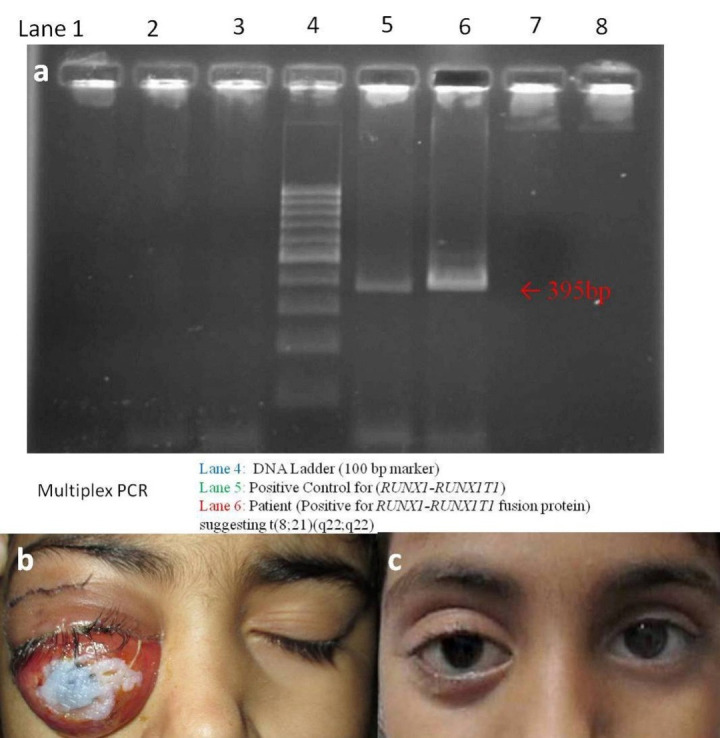
**a** Multiplex PCR showing positive reaction at 395 base pair (red arrow); **b.** Wound healthy, suture intact with increased proptosis, chemosis and cornea protected with antibiotic ointment; **c.** Marked improvement is seen after the completion of chemotherapy

The contrast-enhanced MRI (CE-MRI) orbits showed a well-defined enhancing mass lesion in the right supero-temporal orbit involving the lacrimal gland with a displacement of orbital contents (**Fig. 1c,d**). The right orbital incision biopsy specimen showed tumor cells arranged in diffuse sheets, having round to oval nuclei with fine chromatin, prominent nucleoli, and a moderate amount of cytoplasm (**Fig. 2a**). The IHC showed positivity for CD45, myeloperoxidase, CD34, and CD99, suggestive of myeloid sarcoma (**Fig. 2b,c**).The peripheral blood immune-phenotyping showed AML with aberrant CD56 and CD9 expression. The bone marrow examination showed hypocellular marrow with 25% blasts, and the molecular analysis showed positivity for t (8;21) (AML_ETO1) fusion gene (**Fig. 2d**, **3a**). She was diagnosed to have AML with maturation (AML-M2). Meanwhile, her proptosis increased with severe conjunctival chemosis (**Fig. 3b**) with a diminution of vision OD to 6/ 60.

The pediatric hemato-oncologists started her on injections cytarabine 85 mg (100 mg/ m2), daunorubicin 50 mg (60 mg/ m2), etoposide 85 mg (100 mg/ m2) and triple intrathecal therapy (methotrexate + cytarabine + hydrocortisone). A prompt reduction in OD proptosis and inferior dystopia was noted with marked improvement in ocular movements and her final visual acuity (6/ 9). At 18-months follow-up, she had no features of any ophthalmic or systemic relapse (**Fig. 3d**). 

**Fig. 4:**
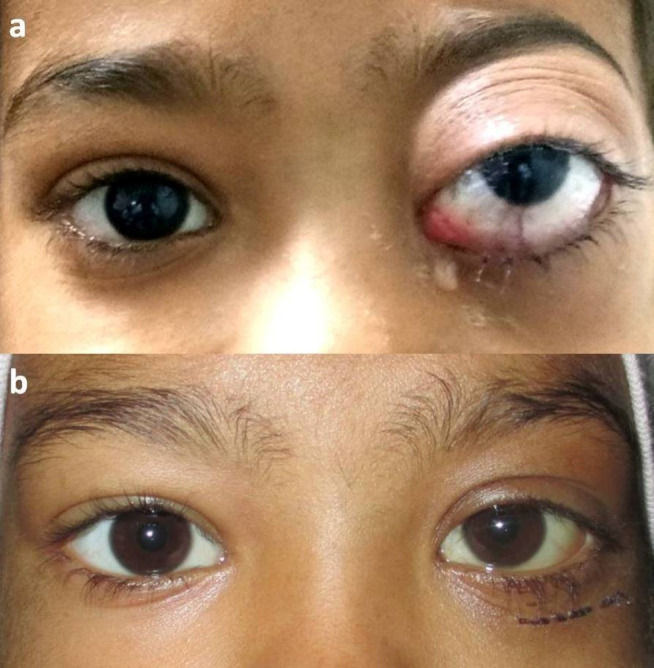
**a** Case 2- OS shows marked proptosis with superior globe displacement and conjunctival congestion; **b.** Significant improvement after 3 months of chemotherapy

*Case 2:* A 8-year-old girl had a rapidly-progressive, painful proptosis OS associated with diminution of vision of 2 months duration. She had no history of malaise, fever, or weight-loss. Ophthalmic examination showed no light perception in the left eye, relative afferent pupillary defect, mild eyelid edema, conjunctival chemosis, and restricted ocular motility in all gazes (**Fig. 4a**). The posterior segment showed disc edema. CEMRI orbits showed an ill-defined, expansile, lytic lesion involving left zygomatic bone, inferior orbital fissure, and left maxillary sinus with intraconal extension causing proptosis.

The incision biopsy showed diffuse sheets of atypical cells with the above-mentioned similar features. The IHC was positive for myeloperoxidase and was negative for CD3, CD20, and cyclin D1. The final histopathological diagnosis of myeloid sarcoma of orbit was established. The systemic workup, including bone-marrow, showed a similar picture. Intravenous chemotherapy injections cytarabine 85 mg (100 mg/ m2), daunorubicin 50 mg (60 mg/ m2), injection etoposide 85 mg (100 mg/ m2) and triple intrathecal therapy (methotrexate + cytarabine + hydrocortisone) was started. A marked improvement in pain and proptosis was noted at the end of the 2-months treatment (**Fig. 4b**). At a follow-up of 15-months, the child was symptom-free and doing well. 

## Discussion

We reported the cases of two girls with unilateral proptosis as the initial presentation of AML. Both were diagnosed as orbital MS after incision biopsies and both responded well to systemic and intrathecal chemotherapy. Myeloid sarcoma constitutes approximately 2.5%-8% of all AML cases [**1**-**5**]. It has been more commonly reported from Asian, African, and Latin American populations, between 6 and 8 years old, with male predominance [**1**-**8**]. On the contrary, both of our patients were females.

Usually, the first presentation of children with AML is easy bruising, fever, joint pain, and weight loss [**3**-**6**]. Ophthalmological features of myeloid sarcoma constitute proptosis, blepharoptosis, lacrimal gland mass, conjunctival mass, and uveitis [**2**,**3**,**6**-**9**]. The proptosis is commonly non-axial due to the proliferation of myeloid blast cells in the extraconal space [**1**,**7**-**9**]. The orbital imaging commonly shows homogenous, irregular soft-tissue mass causing encasement of extraocular muscles and lacrimal gland with lateral orbital predominance [**1**,**7**-**9**].

The diagnosis of myeloid sarcoma may appear concurrently or precede the diagnosis of AML. In patients with proptosis as the initial presentation of leukemia, the bone marrow, and peripheral blood smear involvement occurs within a year [**1**,**4**,**10**]. A retrospective Indian study highlighted the importance of peripheral blood smear testing in similar patients [**2**]. Another Indian study showed that 72.2% of the patients developed systemic manifestations of AML within average of 11.3 months [**9**]. Studies have reported better survival rates for myeloid sarcoma involving CNS and orbit vs. non-CNS myeloid sarcoma [**1**,**2**,**10**].

## Conclusion

In conclusion, myeloid sarcoma (chloroma) in children may present with unilateral, progressive, non-axial proptosis, and orbital imaging with planned incisional biopsy supported by apt histopathology may provide the final diagnosis. Pediatric hematology workup (for AML and other myeloproliferative syndromes) and timely management may provide satisfactory long-term outcomes. 

**Conflict of Interest**

Authors state no conflict of interest.

**Informed Consent and Human and Animal Rights**

Informed consent has been obtained from all individuals included in this study.

**Authorization for the use of human subjects**

Ethical approval: The research related to human use complies with all the relevant national regulations, institutional policies, is in accordance with the tenets of the Helsinki Declaration, and has been approved by the Ethics Committee of Post Graduate Institute of Medical Education and Research, Chandigarh, India.

**Acknowledgments**

None.

**Sources of Funding**

None.

**Disclosures**

None.

**Meeting presentation**

No.

## References

[1] AlSemari MA, Perrotta M, Russo C, Alkatan HM, Maktabi A, Elkhamary S, Crescenzo RMD, Mascolo M, Elefante A, Rombetto L, Capasso R, Strianese D (2020). Orbital myeloid sarcoma (chloroma): Report of 2 cases and literature review. Am J Ophthalmol Case Rep.

[2] Aggarwal E, Mulay K, Honavar SG (2014). Orbital extramedullary granulocytic sarcoma: clinicopathologic correlation with immunohistochemical features. SurvOphthalmol.

[3] Zimmerman LE, Font RL (1975). Ophthalmologic manifestations of granulocytic sarcoma (myeloid sarcoma or chloroma). The third Pan American Association of Ophthalmology and American Journal of Ophthalmology Lecture. Am J Ophthalmol.

[4] Karesh JW, Goldman EJ, Reck K, Kelman SE, Lee EJ, Schiffer CA (1989). A prospective ophthalmic evaluation of patients with acute myeloid leukemia: correlation of ocular and hematologic findings. J Clin Oncol.

[5] Stockl FA, Dolmetsch AM, Saornil MA, Font RL, Burnier MN (1997). Orbital granulocytic sarcoma. Br J Ophthalmol.

[6] Shields JA, Stopyra GA, Marr BP, Shields CL, Pan W, Eagle RC (2003). Bilateral orbital myeloid sarcoma as initial sign of acute myeloid leukemia: case report and review of the literature. Arch Ophthalmol.

[7] Bidar M, Wilson MW, Laquis SJ, Wilson TD, Fleming JC, Wesley RE (2007). Clinical and imaging characteristics of orbital leukemic tumors. OphthalPlastReconstr Surg.

[8] Vera-Aguilera J, Mukarram O, Nutalapati P, Mok M, Bulumulle A, Vera-Aguilera C (2016). Bilateral orbital myeloid sarcoma preceding acute myeloid leukemia in an adult: a case report and review of the literature. J Med Case Rep.

[9] Murthy R, Vemuganti GK, Honavar SG, Naik M, Reddy V (2009). Extramedullary leukemia in children presenting with proptosis. J Hematol Oncol.

[10] Johnston DL, Alonzo TA, Gerbing RB, Lange BJ, Woods WG (2012). Superior outcome of pediatric acute myeloid leukemia patients with orbital and CNS myeloid sarcoma: a report from the Children’s Oncology Group. Pediatr Blood Cancer.

